# Supplementary material for: Signatures of prescribed fire in the microbial communities of Cornus florida are largely undetectable five months post-fire
Source: PeerJ. 2023 Aug 24;11:e15822. doi: 10.7717/peerj.15822 (PMC10460572; doi:10.7717/peerj.15822)
Supplement: Supplemental Information 1 [file peerj-11-15822-s001.docx]

**†** Tree age was estimated by taking a core from the main trunk at breast height for all study trees using an increment borer. Cores were wood glued to 0.15 m plastic pot tags, and then were air dried beneath weight to prevent curling. Once dried, cores were sanded flat and Natural #209 wood stain (Minwax Wood Finish Penetrating Stain, Sherwin-Williams Co., Cleveland, OH, U.S. was thinly applied to provide contrast needed to visualize annular rings using a dissecting microscope.

**Table S.1.** Morphological characteristics of flowering dogwood trees (*Cornus florida*) used in microbial community characterization following prescribed burn. Tree identification information (ID), treatment status (prescribed burn vs. unburned control), tree age, diameter at breast height (DBH), height, and coordinates for all 20 trees.

| **Tree ID** | **Treatment** | **Age estimate (years)†** | **DBH (cm)** | **Height (m)** | **Latitude** | **Longitude** |
| --- | --- | --- | --- | --- | --- | --- |
| 151 | Burned | 39 | 8 | 7.7 | 35.3206765 | -86.1525653 |
| 152 | Burned | 35 | 9 | 9 | 35.3207345 | -86.1526317 |
| 153 | Burned | 37 | 8 | 8 | 35.3205944 | -86.1524959 |
| 154 | Burned | 42 | 8 | 9.4 | 35.3205827 | -86.1527051 |
| 156 | Burned | 29 | 7 | 6.9 | 35.320363 | -86.1525707 |
| 221 | Burned | 32 | 8 | 8.3 | 35.3208499 | -86.1523108 |
| 222 | Burned | 36 | 7 | 6.3 | 35.3208215 | -86.1521985 |
| 223 | Burned | 41 | 7.5 | 8.4 | 35.3205367 | -86.152399 |
| 224 | Burned | 43 | 10 | 8.5 | 35.3205955 | -86.1524862 |
| 225 | Burned | 40 | 7 | 5.4 | 35.3207684 | -86.1526944 |
| 71 | Unburned | 39 | 9 | 8.8 | 35.3204087 | -86.1523903 |
| 72 | Unburned | 38 | 7 | 8.9 | 35.3205077 | -86.1533106 |
| 73 | Unburned | 50 | 8.5 | 9.9 | 35.3203534 | -86.1532791 |
| 74 | Unburned | 44 | 7 | 7.7 | 35.3202281 | -86.1530394 |
| 75 | Unburned | 41 | 8 | 11.4 | 35.3202399 | -86.1528422 |
| 391 | Unburned | 34 | 6 | 7.2 | 35.3203096 | -86.1526387 |
| 392 | Unburned | 39 | 8 | 8.4 | 35.32038 | -86.1523142 |
| 394 | Unburned | 34 | 7 | 7.1 | 35.3201761 | -86.1520654 |
| 396 | Unburned | 39 | 6 | 5.6 | 35.3202738 | -86.1522783 |
| 398 | Unburned | 34 | 6 | 5.6 | 35.3203564 | -86.1525532 |

**Table S.2.** Rarefaction cut-offs and number of samples retained post-rarefaction for each amplicon and each niche of flowering dogwood trees (*Cornus florida*) in the prescribed burn and unburned control treatments. Cut-offs were chosen by reviewing the rarefaction curves in Figure S.1 and S.2 and choosing the sequencing depth that would minimize sample loss and maximize sequencing depth.

| **Amplicon** | **Niche** | **Rarefaction Cut-Off** | **Samples Retained** |
| --- | --- | --- | --- |
| **ITS** | All | 4,000 | 94/100 |
|  | Bulk Soil | 12,151 | 20/20 |
|  | Roots | 1,655 | 17/20 |
|  | Bark | 10,221 | 20/20 |
|  | Stem | 5,098 | 19/20 |
|  | Leaves | 11,293 | 20/20 |
| **16S rRNA** | All | 1034 | 95/100 |
|  | Bulk Soil | 8,488 | 20/20 |
|  | Roots | 3,526 | 20/20 |
|  | Bark | 14,919 | 19/20 |
|  | Stem | 2,640 | 17/20 |
|  | Leaves | 1,034 | 17/20 |

**Table S.3.** Results of post-hoc Tukey’s tests calculated for analysis of variance of Hill numbers (q0 – q2) from plant-associated bacterial/archaeal communities of flowering dogwood (*Cornus florida*) trees. Text in bold represents Hill numbers that significantly differed by prescribed burn treatment (*P*<0.05). Data was log transformed to meet assumptions of ANOVA and post-hoc Tukey’s test.

|  | | | 0D (ASV Richness) | | | | | | | |
| --- | --- | --- | --- | --- | --- | --- | --- | --- | --- | --- |
|  |  |  | Roots | | Bark | | Stem | | Leaves | |
|  |  |  | Prescribed Burn | Unburned Control | Prescribed Burn | Unburned Control | Prescribed Burn | Unburned Control | Prescribed Burn | Unburned Control |
|  |  |  | *P* | *P* | *P* | *P* | *P* | *P* | *P* | *P* |
| Bacteria/Archaea | Roots | Prescribed Burn | -- | **0.02** | **<0.0001** | **<0.0001** | 1.00 | 0.67 | 1.00 | 1.00 |
|  |  | Unburned Control | -- | -- | **0.001** | **<0.0001** | 0.07 | 0.61 | 0.17 | **0.004** |
|  | Bark | Prescribed Burn | -- | -- | -- | 0.99 | **<0.0001** | **<0.0001** | **<0.0001** | **<0.0001** |
|  |  | Unburned Control | -- | -- | -- | -- | **<0.0001** | **<0.0001** | **<0.0001** | **<0.0001** |
|  | Stem | Prescribed Burn | -- | -- | -- | -- | -- | 0.90 | 1.00 | 0.99 |
|  |  | Unburned Control | -- | -- | -- | -- | -- | -- | 0.99 | 0.36 |
|  | Leaves | Prescribed Burn | -- | -- | -- | -- | -- | -- | -- | 0.92 |
|  |  | Unburned Control | -- | -- | -- | -- | -- | -- | -- | -- |
|  | | | 1D (Shannon Entropy) | | | | | | | |
|  |  |  | Roots | | Bark | | Stem | | Leaves | |
|  |  |  | Prescribed Burn | Unburned Control | Prescribed Burn | Unburned Control | Prescribed Burn | Unburned Control | Prescribed Burn | Unburned Control |
|  |  |  | *P* | *P* | *P* | *P* | *P* | *P* | *P* | *P* |
| Bacteria/Archaea | Roots | Prescribed Burn | -- | **0.001** | **<0.0001** | **<0.0001** | 0.19 | **0.012** | 0.08 | 0.65 |
|  |  | Unburned Control | -- | -- | **<0.0001** | **<0.0001** | 0.67 | 0.99 | 0.88 | 0.16 |
|  | Bark | Prescribed Burn | -- | -- | -- | 0.99 | **<0.0001** | **<0.0001** | **<0.0001** | **<0.0001** |
|  |  | Unburned Control | -- | -- | -- | -- | **<0.0001** | **<0.0001** | **<0.0001** | **<0.0001** |
|  | Stem | Prescribed Burn | -- | -- | -- | -- | -- | 0.99 | 1.00 | 0.99 |
|  |  | Unburned Control | -- | -- | -- | -- | -- | -- | 1.00 | 0.63 |
|  | Leaves | Prescribed Burn | -- | -- | -- | -- | -- | -- | -- | 0.93 |
|  |  | Unburned Control | -- | -- | -- | -- | -- | -- | -- | -- |
|  | | | 2D (Inverse Simpson) | | | | | | | |
|  |  |  | Roots | | Bark | | Stem | | Leaves | |
|  |  |  | Prescribed Burn | Unburned Control | Prescribed Burn | Unburned Control | Prescribed Burn | Unburned Control | Prescribed Burn | Unburned Control |
|  |  |  | *P* | *P* | *P* | *P* | *P* | *P* | *P* | *P* |
| Bacteria/Archaea | Roots | Prescribed Burn | -- | **0.03** | **<0.0001** | **<0.0001** | 0.12 | **0.02** | 0.06 | 0.55 |
|  |  | Unburned Control | -- | -- | **<0.0001** | **<0.0001** | 1.00 | 1.00 | 1.00 | 0.90 |
|  | Bark | Prescribed Burn | -- | -- | -- | 1.00 | **<0.0001** | **<0.0001** | **<0.0001** | **<0.0001** |
|  |  | Unburned Control | -- | -- | -- | -- | **<0.0001** | **<0.0001** | **<0.0001** | **<0.0001** |
|  | Stem | Prescribed Burn | -- | -- | -- | -- | -- | 1.00 | 1.00 | 0.98 |
|  |  | Unburned Control | -- | -- | -- | -- | -- | -- | 1.00 | 0.83 |
|  | Leaves | Prescribed Burn | -- | -- | -- | -- | -- | -- | -- | 0.94 |
|  |  | Unburned Control | -- | -- | -- | -- | -- | -- | -- | -- |

**Table S.4.** Results of DESeq2 analysis of bacterial/archaeal communities associated with the roots flowering dogwood (*Cornus florida*) roots in prescribed burn and unburned control plots. The log2fold changes represent changes in abundance in prescribed burn treated roots compared to unburned control roots.

| ASV ID | Taxonomy | baseMean | log2FoldChange | lfcSE | stat | *P* | *P-adj* |
| --- | --- | --- | --- | --- | --- | --- | --- |
| asv_16s 10368 | K_Bacteria;P_Proteobacteria;C_Alphaproteobacteria;  O_Acetobacterales;F_Acetobacteraceae;G_NA;s_NA | 5.01 | -2.95 | 0.89 | -3.33 | 0.00086 | 0.00520 |
| asv_16s 10389 | K_Bacteria;P_Proteobacteria;C_Alphaproteobacteria;  O_Rhodospirillales;F_Magnetospirillaceae;  G_Telmatospirillum;s_NA | 4.18 | 2.66 | 0.88 | 3.02 | 0.00255 | 0.01010 |
| asv_16s 10441 | K_Bacteria;P_Proteobacteria;C_Alphaproteobacteria;O_NA;  F_NA;G_NA;s_NA | 6.84 | -3.45 | 0.94 | -3.67 | 0.00024 | 0.00223 |
| asv_16s 10443 | K_Bacteria;P_Proteobacteria;C_Alphaproteobacteria;  O_Elsterales;F_NA;G_NA;s_NA | 6.73 | -3.42 | 0.92 | -3.70 | 0.00021 | 0.00206 |
| asv_16s 10446 | K_Bacteria;P_Proteobacteria;C_Alphaproteobacteria;  O_Elsterales;F_NA;G_NA;s_NA | 4.88 | 2.92 | 0.88 | 3.30 | 0.00097 | 0.00559 |
| asv_16s 10511 | K_Bacteria;P_Proteobacteria;C_Alphaproteobacteria;  O_NA;F_NA;G_NA;s_NA | 4.48 | 2.77 | 0.94 | 2.96 | 0.00312 | 0.01126 |
| asv_16s 10709 | K_Bacteria;P_Myxococcota;C_Polyangia;O_Haliangiales;  F_Haliangiaceae;G_Haliangium;s_NA | 9.04 | -3.88 | 0.97 | -4.01 | 0.00006 | 0.00093 |
| asv_16s 10739 | K_Bacteria;P_Proteobacteria;C_Alphaproteobacteria;  O_Rhizobiales;F_Rhizobiaceae;G_Allorhizobium-Neorhizobium-Pararhizobium-Rhizobium;s_NA | 12.50 | -4.38 | 1.10 | -3.96 | 0.00007 | 0.00098 |
| asv_16s 10775 | K_Bacteria;P_Proteobacteria;C_Alphaproteobacteria;  O_Rhizobiales;F_Rhizobiaceae;G_Allorhizobium-Neorhizobium-Pararhizobium-Rhizobium;s_phaseoli | 7.36 | -3.56 | 1.00 | -3.57 | 0.00036 | 0.00282 |
| asv_16s 10824 | K_Bacteria;P_Proteobacteria;C_Alphaproteobacteria;  O_Rhizobiales;F_Xanthobacteraceae;G_Bradyrhizobium;s_NA | 8.16 | 3.73 | 1.03 | 3.64 | 0.00027 | 0.00226 |
| asv_16s 10844 | K_Bacteria;P_Proteobacteria;C_Alphaproteobacteria;  O_Rhizobiales;F_Rhizobiaceae;G_Allorhizobium-Neorhizobium-Pararhizobium-Rhizobium;s_NA | 4.17 | -2.65 | 0.93 | -2.86 | 0.00425 | 0.01366 |
| asv_16s 11113 | K_Bacteria;P_Proteobacteria;C_Alphaproteobacteria;  O_Rhizobiales;F_Beijerinckiaceae;G_Roseiarcus;s_NA | 9.14 | -3.90 | 0.97 | -4.02 | 0.00006 | 0.00093 |
| asv_16s 11122 | K_Bacteria;P_Proteobacteria;C_Alphaproteobacteria;  O_Rhizobiales;F_Labraceae;G_Labrys;s_miyagiensis | 7.81 | -2.31 | 0.90 | -2.56 | 0.01046 | 0.02774 |
| asv_16s 11199 | K_Bacteria;P_Proteobacteria;C_Alphaproteobacteria;  O_Rhizobiales;F_Xanthobacteraceae;G_NA;s_NA | 3.73 | -2.46 | 0.90 | -2.72 | 0.00651 | 0.01832 |
| asv_16s 11399 | K_Bacteria;P_Proteobacteria;C_Alphaproteobacteria;  O_Acetobacterales;F_Acetobacteraceae;G_Acidocella;s_NA | 10.79 | -2.77 | 0.96 | -2.88 | 0.00399 | 0.01326 |
| asv_16s 11402 | K_Bacteria;P_Proteobacteria;C_Alphaproteobacteria;  O_Acetobacterales;F_Acetobacteraceae;G_Acidocella;s_NA | 3.83 | -2.50 | 0.91 | -2.75 | 0.00589 | 0.01719 |
| asv_16s 11403 | K_Bacteria;P_Proteobacteria;C_Alphaproteobacteria;  O_Acetobacterales;F_Acetobacteraceae;G_Acidocella;s_NA | 12.96 | -4.43 | 1.03 | -4.32 | 0.00002 | 0.00035 |
| asv_16s 11456 | K_Bacteria;P_Proteobacteria;C_Gammaproteobacteria;  O_WD260;F_NA;G_NA;s_NA | 7.58 | 3.62 | 0.94 | 3.84 | 0.00012 | 0.00130 |
| asv_16s 11480 | K_Bacteria;P_Proteobacteria;C_Gammaproteobacteria;O_WD260;F_NA;G_NA;s_NA | 15.29 | -3.21 | 0.94 | -3.42 | 0.00063 | 0.00425 |
| asv_16s 11500 | K_Bacteria;P_Proteobacteria;C_Gammaproteobacteria;O_WD260;F_NA;G_NA;s_NA | 9.13 | 3.91 | 0.99 | 3.96 | 0.00007 | 0.00098 |
| asv_16s 11504 | K_Bacteria;P_Proteobacteria;C_Gammaproteobacteria;O_WD260;F_NA;G_NA;s_NA | 4.38 | -2.73 | 0.89 | -3.06 | 0.00219 | 0.00906 |
| asv_16s 11517 | K_Bacteria;P_Proteobacteria;C_Gammaproteobacteria;O_WD260;F_NA;G_NA;s_NA | 4.15 | -2.64 | 0.88 | -2.99 | 0.00277 | 0.01041 |
| asv_16s 11599 | K_Bacteria;P_Proteobacteria;C_Gammaproteobacteria;O_Gammaproteobacteria Incertae Sedis;F_Unknown Family;G_Acidibacter;s_NA | 67.32 | 3.71 | 1.19 | 3.13 | 0.00177 | 0.00798 |
| asv_16s 11613 | K_Bacteria;P_Proteobacteria;C_Gammaproteobacteria;O_Gammaproteobacteria Incertae Sedis;F_Unknown Family;G_Acidibacter;s_NA | 65.05 | 3.29 | 1.19 | 2.77 | 0.00560 | 0.01652 |
| asv_16s 11620 | K_Bacteria;P_Proteobacteria;C_Gammaproteobacteria;O_Gammaproteobacteria Incertae Sedis;F_Unknown Family;G_Acidibacter;s_NA | 22.38 | -2.74 | 1.07 | -2.55 | 0.01092 | 0.02869 |
| asv_16s 11667 | K_Bacteria;P_Proteobacteria;C_Gammaproteobacteria;O_Gammaproteobacteria Incertae Sedis;F_Unknown Family;G_Acidibacter;s_NA | 4.26 | -2.68 | 0.96 | -2.81 | 0.00497 | 0.01546 |
| asv_16s 11838 | K_Bacteria;P_Proteobacteria;C_Alphaproteobacteria;O_Rhizobiales;F_Xanthobacteraceae;G_Bradyrhizobium;s_japonicum | 36.99 | -2.64 | 1.11 | -2.38 | 0.01749 | 0.04284 |
| asv_16s 11965 | K_Bacteria;P_Proteobacteria;C_Gammaproteobacteria;O_Gammaproteobacteria Incertae Sedis;F_Unknown Family;G_Acidibacter;s_NA | 6.98 | -3.33 | 0.92 | -3.63 | 0.00028 | 0.00226 |
| asv_16s 11968 | K_Bacteria;P_Proteobacteria;C_Gammaproteobacteria;O_Gammaproteobacteria Incertae Sedis;F_Unknown Family;G_Acidibacter;s_NA | 13.04 | -4.44 | 0.98 | -4.54 | 0.00001 | 0.00019 |
| asv_16s 11973 | K_Bacteria;P_Proteobacteria;C_Gammaproteobacteria;O_Gammaproteobacteria Incertae Sedis;F_Unknown Family;G_Acidibacter;s_NA | 4.05 | -2.60 | 0.85 | -3.07 | 0.00211 | 0.00898 |
| asv_16s 12021 | K_Bacteria;P_Proteobacteria;C_Gammaproteobacteria;O_Diplorickettsiales;F_Diplorickettsiaceae;G_NA;s_NA | 6.09 | 2.40 | 0.90 | 2.66 | 0.00791 | 0.02137 |
| asv_16s 12240 | K_Bacteria;P_Acidobacteriota;C_Acidobacteriae;O_Subgroup 2;F_NA;G_NA;s_NA | 15.48 | -3.23 | 0.89 | -3.64 | 0.00027 | 0.00226 |
| asv_16s 12280 | K_Bacteria;P_Acidobacteriota;C_Acidobacteriae;O_Bryobacterales;F_Bryobacteraceae;G_Bryobacter;s_NA | 24.31 | -2.34 | 0.97 | -2.41 | 0.01608 | 0.03998 |
| asv_16s 12481 | K_Bacteria;P_Acidobacteriota;C_Acidobacteriae;O_Subgroup 2;F_NA;G_NA;s_NA | 4.05 | -2.60 | 0.85 | -3.08 | 0.00210 | 0.00898 |
| asv_16s 12503 | K_Bacteria;P_Acidobacteriota;C_Acidobacteriae;O_Subgroup 2;F_NA;G_NA;s_NA | 27.14 | -3.19 | 0.98 | -3.25 | 0.00114 | 0.00624 |
| asv_16s 12589 | K_Bacteria;P_Acidobacteriota;C_Acidobacteriae;O_Bryobacterales;F_Bryobacteraceae;G_Bryobacter;s_NA | 20.95 | 2.87 | 0.96 | 2.99 | 0.00275 | 0.01041 |
| asv_16s 13058 | K_Bacteria;P_Actinobacteriota;C_Thermoleophilia;O_Solirubrobacterales;F_67-14;G_NA;s_NA | 5.92 | -3.22 | 0.91 | -3.53 | 0.00041 | 0.00303 |
| asv_16s 13607 | K_Bacteria;P_Proteobacteria;C_Alphaproteobacteria;O_Caulobacterales;F_Caulobacteraceae;G_Caulobacter;s_NA | 3.88 | -2.53 | 0.91 | -2.77 | 0.00557 | 0.01652 |
| asv_16s 14402 | K_Bacteria;P_Firmicutes;C_Bacilli;O_Entomoplasmatales;F_type III;G_NA;s_NA | 7.13 | -3.51 | 0.99 | -3.55 | 0.00039 | 0.00297 |
| asv_16s 14429 | K_Bacteria;P_Cyanobacteria;C_Vampirivibrionia;O_Obscuribacterales;F_Obscuribacteraceae;G_NA;s_NA | 4.57 | 2.81 | 0.90 | 3.13 | 0.00177 | 0.00798 |
| asv_16s 14690 | K_Bacteria;P_Acidobacteriota;C_Acidobacteriae;O_Acidobacteriales;F_Acidobacteriaceae (Subgroup 1);G_Granulicella;s_NA | 8.78 | 2.30 | 0.94 | 2.44 | 0.01450 | 0.03645 |
| asv_16s 14692 | K_Bacteria;P_Acidobacteriota;C_Acidobacteriae;O_Acidobacteriales;F_Acidobacteriaceae (Subgroup 1);G_Granulicella;s_NA | 3.94 | -2.55 | 0.91 | -2.79 | 0.00527 | 0.01602 |
| asv_16s 14731 | K_Bacteria;P_Acidobacteriota;C_Acidobacteriae;O_Acidobacteriales;F_Acidobacteriaceae (Subgroup 1);G_Granulicella;s_NA | 12.45 | -2.29 | 0.95 | -2.40 | 0.01619 | 0.03998 |
| asv_16s 14781 | K_Bacteria;P_Acidobacteriota;C_Acidobacteriae;O_Acidobacteriales;F_NA;G_NA;s_NA | 3.72 | 2.46 | 0.86 | 2.86 | 0.00417 | 0.01354 |
| asv_16s 14797 | K_Bacteria;P_Acidobacteriota;C_Acidobacteriae;O_Acidobacteriales;F_NA;G_NA;s_NA | 3.77 | -2.48 | 0.91 | -2.74 | 0.00623 | 0.01801 |
| asv_16s 14827 | K_Bacteria;P_Acidobacteriota;C_Acidobacteriae;O_Acidobacteriales;F_Acidobacteriaceae (Subgroup 1);G_Granulicella;s_NA | 3.70 | -2.45 | 0.86 | -2.85 | 0.00440 | 0.01398 |
| asv_16s 1488 | K_Bacteria;P_Bacteroidota;C_Bacteroidia;O_Chitinophagales;F_Chitinophagaceae;G_Puia;s_NA | 26.74 | -5.37 | 1.03 | -5.19 | 0.00000 | 0.00004 |
| asv_16s 14933 | K_Bacteria;P_Acidobacteriota;C_Acidobacteriae;O_Acidobacteriales;F_Acidobacteriaceae (Subgroup 1);G_Acidipila-Silvibacterium;s_NA | 25.03 | -2.86 | 0.91 | -3.16 | 0.00159 | 0.00755 |
| asv_16s 14939 | K_Bacteria;P_Acidobacteriota;C_Acidobacteriae;O_Acidobacteriales;F_Acidobacteriaceae (Subgroup 1);G_Acidipila-Silvibacterium;s_NA | 4.01 | 2.59 | 0.87 | 2.96 | 0.00303 | 0.01118 |
| asv_16s 14975 | K_Bacteria;P_Acidobacteriota;C_Acidobacteriae;O_Acidobacteriales;F_Acidobacteriaceae (Subgroup 1);G_NA;s_NA | 4.33 | -2.71 | 0.85 | -3.18 | 0.00148 | 0.00724 |
| asv_16s 15007 | K_Bacteria;P_Acidobacteriota;C_Acidobacteriae;O_Acidobacteriales;F_Acidobacteriaceae (Subgroup 1);G_Acidipila-Silvibacterium;s_NA | 10.32 | -2.27 | 0.93 | -2.45 | 0.01435 | 0.03637 |
| asv_16s 15042 | K_Bacteria;P_Proteobacteria;C_Gammaproteobacteria;O_Burkholderiales;F_Comamonadaceae;G_NA;s_NA | 3.83 | 2.51 | 0.86 | 2.91 | 0.00367 | 0.01263 |
| asv_16s 15076 | K_Bacteria;P_Proteobacteria;C_Gammaproteobacteria;O_Burkholderiales;F_Burkholderiaceae;G_Burkholderia-Caballeronia-Paraburkholderia;s_NA | 102.97 | -4.91 | 0.96 | -5.14 | 0.00000 | 0.00004 |
| asv_16s 1515 | K_Bacteria;P_Bacteroidota;C_Bacteroidia;O_Chitinophagales;F_Chitinophagaceae;G_Puia;s_NA | 5.34 | 3.06 | 0.90 | 3.40 | 0.00069 | 0.00448 |
| asv_16s 1522 | K_Bacteria;P_Bacteroidota;C_Bacteroidia;O_Chitinophagales;F_Chitinophagaceae;G_Puia;s_NA | 6.13 | 3.28 | 0.98 | 3.35 | 0.00080 | 0.00495 |
| asv_16s 15261 | K_Bacteria;P_Proteobacteria;C_Gammaproteobacteria;O_Burkholderiales;F_Burkholderiaceae;G_Burkholderia-Caballeronia-Paraburkholderia;s_phenazinium | 37.85 | -3.10 | 1.05 | -2.96 | 0.00306 | 0.01118 |
| asv_16s 15265 | K_Bacteria;P_Proteobacteria;C_Gammaproteobacteria;O_Burkholderiales;F_Burkholderiaceae;G_Burkholderia-Caballeronia-Paraburkholderia;s_NA | 4.58 | -2.80 | 0.83 | -3.39 | 0.00070 | 0.00448 |
| asv_16s 15468 | K_Bacteria;P_Proteobacteria;C_Gammaproteobacteria;O_Burkholderiales;F_Burkholderiaceae;G_Burkholderia-Caballeronia-Paraburkholderia;s_NA | 14.74 | -3.41 | 1.03 | -3.32 | 0.00090 | 0.00531 |
| asv_16s 15872 | K_Bacteria;P_Firmicutes;C_Bacilli;O_Entomoplasmatales;F_type III;G_NA;s_NA | 20.59 | -5.12 | 1.08 | -4.74 | 0.00000 | 0.00012 |
| asv_16s 1594 | K_Bacteria;P_Bacteroidota;C_Bacteroidia;O_Chitinophagales;F_Chitinophagaceae;G_Puia;s_NA | 3.71 | -2.45 | 0.90 | -2.72 | 0.00660 | 0.01832 |
| asv_16s 16063 | K_Bacteria;P_Proteobacteria;C_Alphaproteobacteria;O_Caulobacterales;F_Caulobacteraceae;G_NA;s_NA | 4.43 | -2.75 | 0.89 | -3.08 | 0.00210 | 0.00898 |
| asv_16s 2474 | K_Bacteria;P_Actinobacteriota;C_Actinobacteria;O_Frankiales;F_Acidothermaceae;G_Acidothermus;s_NA | 4.16 | -2.64 | 0.82 | -3.24 | 0.00121 | 0.00647 |
| asv_16s 2652 | K_Bacteria;P_Proteobacteria;C_Gammaproteobacteria;O_JG36-TzT-191;F_NA;G_NA;s_NA | 5.02 | -2.06 | 0.82 | -2.50 | 0.01254 | 0.03265 |
| asv_16s 2653 | K_Bacteria;P_Proteobacteria;C_Gammaproteobacteria;O_JG36-TzT-191;F_NA;G_NA;s_NA | 4.68 | -2.84 | 0.86 | -3.28 | 0.00103 | 0.00584 |
| asv_16s 3232 | K_Bacteria;P_Firmicutes;C_Bacilli;O_Bacillales;F_Bacillaceae;G_Bacillus;s_NA | 5.51 | 3.11 | 0.90 | 3.47 | 0.00051 | 0.00371 |
| asv_16s 3980 | K_Bacteria;P_Verrucomicrobiota;C_Chlamydiae;O_Chlamydiales;F_Parachlamydiaceae;G_Candidatus Protochlamydia;s_NA | 9.91 | -4.02 | 1.00 | -4.03 | 0.00006 | 0.00093 |
| asv_16s 4305 | K_Bacteria;P_Planctomycetota;C_Planctomycetes;O_Isosphaerales;F_Isosphaeraceae;G_NA;s_NA | 7.00 | -2.35 | 0.91 | -2.57 | 0.01014 | 0.02712 |
| asv_16s 4355 | K_Bacteria;P_Planctomycetota;C_Planctomycetes;O_Isosphaerales;F_Isosphaeraceae;G_Tundrisphaera;s_NA | 3.70 | -2.45 | 0.90 | -2.71 | 0.00666 | 0.01832 |
| asv_16s 4379 | K_Bacteria;P_Planctomycetota;C_Planctomycetes;O_Isosphaerales;F_Isosphaeraceae;G_Tundrisphaera;s_NA | 4.25 | -2.68 | 0.93 | -2.88 | 0.00398 | 0.01326 |
| asv_16s 4381 | K_Bacteria;P_Planctomycetota;C_Planctomycetes;O_Isosphaerales;F_Isosphaeraceae;G_Aquisphaera;s_NA | 6.92 | -3.46 | 0.89 | -3.90 | 0.00010 | 0.00115 |
| asv_16s 4433 | K_Bacteria;P_Planctomycetota;C_Planctomycetes;O_Isosphaerales;F_Isosphaeraceae;G_Tundrisphaera;s_NA | 4.05 | -2.60 | 0.89 | -2.91 | 0.00356 | 0.01255 |
| asv_16s 4447 | K_Bacteria;P_Planctomycetota;C_Planctomycetes;O_Isosphaerales;F_Isosphaeraceae;G_Tundrisphaera;s_NA | 4.73 | -2.85 | 0.95 | -3.01 | 0.00264 | 0.01018 |
| asv_16s 4457 | K_Bacteria;P_Planctomycetota;C_Planctomycetes;O_Isosphaerales;F_Isosphaeraceae;G_Aquisphaera;s_NA | 16.13 | -2.55 | 0.94 | -2.72 | 0.00659 | 0.01832 |
| asv_16s 4463 | K_Bacteria;P_Planctomycetota;C_Planctomycetes;O_Isosphaerales;F_Isosphaeraceae;G_Aquisphaera;s_NA | 12.14 | -4.33 | 0.99 | -4.39 | 0.00001 | 0.00027 |
| asv_16s 4464 | K_Bacteria;P_Planctomycetota;C_Planctomycetes;O_Isosphaerales;F_Isosphaeraceae;G_Aquisphaera;s_NA | 3.94 | -2.55 | 0.87 | -2.92 | 0.00346 | 0.01236 |
| asv_16s 4471 | K_Bacteria;P_Planctomycetota;C_Planctomycetes;O_Isosphaerales;F_Isosphaeraceae;G_Aquisphaera;s_NA | 6.66 | -2.35 | 0.84 | -2.82 | 0.00483 | 0.01518 |
| asv_16s 4475 | K_Bacteria;P_Planctomycetota;C_Planctomycetes;O_Isosphaerales;F_Isosphaeraceae;G_Aquisphaera;s_NA | 3.53 | -2.36 | 0.85 | -2.79 | 0.00534 | 0.01607 |
| asv_16s 4850 | K_Bacteria;P_Verrucomicrobiota;C_Verrucomicrobiae;O_Methylacidiphilales;F_Methylacidiphilaceae;G_NA;s_NA | 4.83 | -2.89 | 0.84 | -3.42 | 0.00062 | 0.00425 |
| asv_16s 4878 | K_Bacteria;P_Verrucomicrobiota;C_Verrucomicrobiae;O_Methylacidiphilales;F_Methylacidiphilaceae;G_NA;s_NA | 8.40 | -3.77 | 0.96 | -3.91 | 0.00009 | 0.00114 |
| asv_16s 5443 | K_Bacteria;P_Verrucomicrobiota;C_Verrucomicrobiae;O_Chthoniobacterales;F_Xiphinematobacteraceae;G_Candidatus Xiphinematobacter;s_NA | 9.88 | -4.02 | 1.00 | -4.03 | 0.00005 | 0.00093 |
| asv_16s 5617 | K_Bacteria;P_Verrucomicrobiota;C_Verrucomicrobiae;O_Chthoniobacterales;F_Chthoniobacteraceae;G_Chthoniobacter;s_NA | 3.87 | -2.52 | 0.87 | -2.90 | 0.00372 | 0.01264 |
| asv_16s 6052 | K_Bacteria;P_Verrucomicrobiota;C_Verrucomicrobiae;O_Chthoniobacterales;F_Xiphinematobacteraceae;G_Candidatus Xiphinematobacter;s_NA | 13.90 | -2.13 | 0.86 | -2.49 | 0.01276 | 0.03292 |
| asv_16s 6518 | K_Bacteria;P_Planctomycetota;C_Planctomycetes;O_Gemmatales;F_Gemmataceae;G_Gemmata;s_NA | 3.88 | -2.70 | 0.90 | -3.01 | 0.00259 | 0.01010 |
| asv_16s 6527 | K_Bacteria;P_Planctomycetota;C_Planctomycetes;O_Gemmatales;F_Gemmataceae;G_Gemmata;s_NA | 5.17 | 3.01 | 0.92 | 3.27 | 0.00109 | 0.00603 |
| asv_16s 6647 | K_Bacteria;P_Planctomycetota;C_Planctomycetes;O_Gemmatales;F_Gemmataceae;G_Gemmata;s_NA | 9.93 | -4.03 | 0.90 | -4.45 | 0.00001 | 0.00023 |
| asv_16s 6893 | K_Bacteria;P_Planctomycetota;C_Planctomycetes;O_Gemmatales;F_Gemmataceae;G_NA;s_NA | 3.73 | 2.65 | 0.91 | 2.91 | 0.00364 | 0.01263 |
| asv_16s 8170 | K_Bacteria;P_WPS-2;C_NA;O_NA;F_NA;G_NA;s_NA | 3.76 | -2.47 | 0.86 | -2.87 | 0.00412 | 0.01354 |
| asv_16s 8273 | K_Bacteria;P_Firmicutes;C_Bacilli;O_Entomoplasmatales;F_type III;G_NA;s_NA | 6.74 | -3.42 | 1.01 | -3.38 | 0.00071 | 0.00448 |
| asv_16s 8277 | K_Bacteria;P_Firmicutes;C_Bacilli;O_Entomoplasmatales;F_type III;G_NA;s_NA | 14.43 | -4.59 | 1.03 | -4.45 | 0.00001 | 0.00023 |
| asv_16s 8330 | K_Bacteria;P_Chloroflexi;C_Ktedonobacteria;O_Ktedonobacterales;F_Ktedonobacteraceae;G_Thermosporothrix;s_NA | 19.04 | 5.02 | 1.07 | 4.67 | 0.00000 | 0.00012 |
| asv_16s 8369 | K_Bacteria;P_Chloroflexi;C_Ktedonobacteria;O_Ktedonobacterales;F_Ktedonobacteraceae;G_Thermosporothrix;s_NA | 5.46 | 3.10 | 0.90 | 3.44 | 0.00059 | 0.00416 |
| asv_16s 8375 | K_Bacteria;P_Chloroflexi;C_Ktedonobacteria;O_Ktedonobacterales;F_Ktedonobacteraceae;G_NA;s_NA | 4.36 | -2.72 | 0.87 | -3.14 | 0.00170 | 0.00793 |
| asv_16s 8507 | K_Bacteria;P_Firmicutes;C_Bacilli;O_NA;F_NA;G_NA;s_NA | 4.97 | -2.50 | 0.90 | -2.79 | 0.00526 | 0.01602 |
| asv_16s 8774 | K_Bacteria;P_Actinobacteriota;C_Actinobacteria;O_Pseudonocardiales;F_Pseudonocardiaceae;G_Amycolatopsis;s_NA | 23.95 | -5.35 | 1.14 | -4.68 | 0.00000 | 0.00012 |
| asv_16s 8775 | K_Bacteria;P_Actinobacteriota;C_Actinobacteria;O_Pseudonocardiales;F_Pseudonocardiaceae;G_NA;s_NA | 7.67 | -3.63 | 0.99 | -3.66 | 0.00025 | 0.00226 |
| asv_16s 8811 | K_Bacteria;P_Actinobacteriota;C_Actinobacteria;O_Corynebacteriales;F_Mycobacteriaceae;G_Mycobacterium;s_NA | 4.90 | -2.91 | 0.91 | -3.19 | 0.00140 | 0.00720 |
| asv_16s 8825 | K_Bacteria;P_Actinobacteriota;C_Actinobacteria;O_Corynebacteriales;F_Mycobacteriaceae;G_Mycobacterium;s_NA | 8.18 | -3.73 | 0.96 | -3.88 | 0.00010 | 0.00119 |
| asv_16s 8844 | K_Bacteria;P_Actinobacteriota;C_Actinobacteria;O_Corynebacteriales;F_Mycobacteriaceae;G_Mycobacterium;s_NA | 4.80 | -2.88 | 0.95 | -3.02 | 0.00250 | 0.01005 |
| asv_16s 8865 | K_Bacteria;P_Actinobacteriota;C_Actinobacteria;O_Corynebacteriales;F_Mycobacteriaceae;G_Mycobacterium;s_NA | 6.35 | -3.33 | 0.92 | -3.63 | 0.00028 | 0.00226 |
| asv_16s 8981 | K_Bacteria;P_Actinobacteriota;C_Actinobacteria;O_Frankiales;F_Acidothermaceae;G_Acidothermus;s_NA | 5.46 | 3.10 | 0.97 | 3.19 | 0.00142 | 0.00720 |
| asv_16s 8999 | K_Bacteria;P_Actinobacteriota;C_Actinobacteria;O_Frankiales;F_Acidothermaceae;G_Acidothermus;s_NA | 625.73 | 3.90 | 1.45 | 2.69 | 0.00705 | 0.01921 |
| asv_16s 9003 | K_Bacteria;P_Actinobacteriota;C_Actinobacteria;O_Frankiales;F_Acidothermaceae;G_Acidothermus;s_NA | 4.91 | -2.92 | 0.96 | -3.05 | 0.00229 | 0.00933 |
| asv_16s 9018 | K_Bacteria;P_Actinobacteriota;C_Actinobacteria;O_Frankiales;F_Acidothermaceae;G_Acidothermus;s_NA | 21.36 | 2.60 | 1.05 | 2.48 | 0.01318 | 0.03371 |
| asv_16s 9026 | K_Bacteria;P_Actinobacteriota;C_Actinobacteria;O_Frankiales;F_Acidothermaceae;G_Acidothermus;s_NA | 16.24 | 4.78 | 1.02 | 4.68 | 0.00000 | 0.00012 |
| asv_16s 9219 | K_Bacteria;P_Actinobacteriota;C_Actinobacteria;O_Frankiales;F_Acidothermaceae;G_Acidothermus;s_NA | 12.26 | 3.96 | 0.99 | 4.02 | 0.00006 | 0.00093 |
| asv_16s 9221 | K_Bacteria;P_Actinobacteriota;C_Actinobacteria;O_Frankiales;F_Acidothermaceae;G_Acidothermus;s_NA | 8.10 | -3.71 | 0.96 | -3.86 | 0.00011 | 0.00124 |
| asv_16s 9305 | K_Bacteria;P_Actinobacteriota;C_Actinobacteria;O_Frankiales;F_Acidothermaceae;G_Acidothermus;s_NA | 6.73 | -3.42 | 0.90 | -3.80 | 0.00014 | 0.00144 |
| asv_16s 9313 | K_Bacteria;P_Actinobacteriota;C_Actinobacteria;O_Frankiales;F_Acidothermaceae;G_Acidothermus;s_NA | 29.48 | -3.41 | 1.08 | -3.16 | 0.00159 | 0.00755 |
| asv_16s 9316 | K_Bacteria;P_Actinobacteriota;C_Actinobacteria;O_Frankiales;F_Acidothermaceae;G_Acidothermus;s_NA | 4.62 | -2.99 | 0.93 | -3.22 | 0.00130 | 0.00682 |
| asv_16s 9398 | K_Bacteria;P_Actinobacteriota;C_Actinobacteria;O_Frankiales;F_Acidothermaceae;G_Acidothermus;s_NA | 15.99 | 4.76 | 1.05 | 4.52 | 0.00001 | 0.00019 |
| asv_16s 9406 | K_Bacteria;P_Actinobacteriota;C_Actinobacteria;O_Frankiales;F_Acidothermaceae;G_Acidothermus;s_NA | 8.93 | -3.86 | 0.97 | -3.98 | 0.00007 | 0.00098 |
| asv_16s 9421 | K_Bacteria;P_Actinobacteriota;C_Actinobacteria;O_Frankiales;F_Acidothermaceae;G_Acidothermus;s_NA | 28.16 | -5.59 | 1.11 | -5.02 | 0.00000 | 0.00005 |
| asv_16s 9424 | K_Bacteria;P_Actinobacteriota;C_Actinobacteria;O_Frankiales;F_Acidothermaceae;G_Acidothermus;s_NA | 4.51 | -2.78 | 0.90 | -3.10 | 0.00195 | 0.00869 |
| asv_16s 9434 | K_Bacteria;P_Actinobacteriota;C_Actinobacteria;O_Frankiales;F_Acidothermaceae;G_Acidothermus;s_NA | 4.35 | 2.73 | 0.89 | 3.07 | 0.00217 | 0.00906 |
| asv_16s 9461 | K_Bacteria;P_Actinobacteriota;C_Actinobacteria;O_Frankiales;F_Acidothermaceae;G_Acidothermus;s_NA | 19.67 | -3.80 | 0.99 | -3.82 | 0.00013 | 0.00137 |
| asv_16s 9596 | K_Bacteria;P_Actinobacteriota;C_Actinobacteria;O_Frankiales;F_Acidothermaceae;G_Acidothermus;s_NA | 5.56 | -3.12 | 0.98 | -3.19 | 0.00145 | 0.00721 |
| asv_16s 9773 | K_Bacteria;P_Proteobacteria;C_Alphaproteobacteria;O_Rhodospirillales;F_Magnetospirillaceae;G_NA;s_NA | 13.36 | 4.49 | 1.11 | 4.03 | 0.00006 | 0.00093 |
| asv_16s 9938 | K_Bacteria;P_Proteobacteria;C_Alphaproteobacteria;O_Sphingomonadales;F_Sphingomonadaceae;G_Sphingomonas;s_NA | 3.71 | -2.45 | 0.90 | -2.72 | 0.00660 | 0.01832 |

**
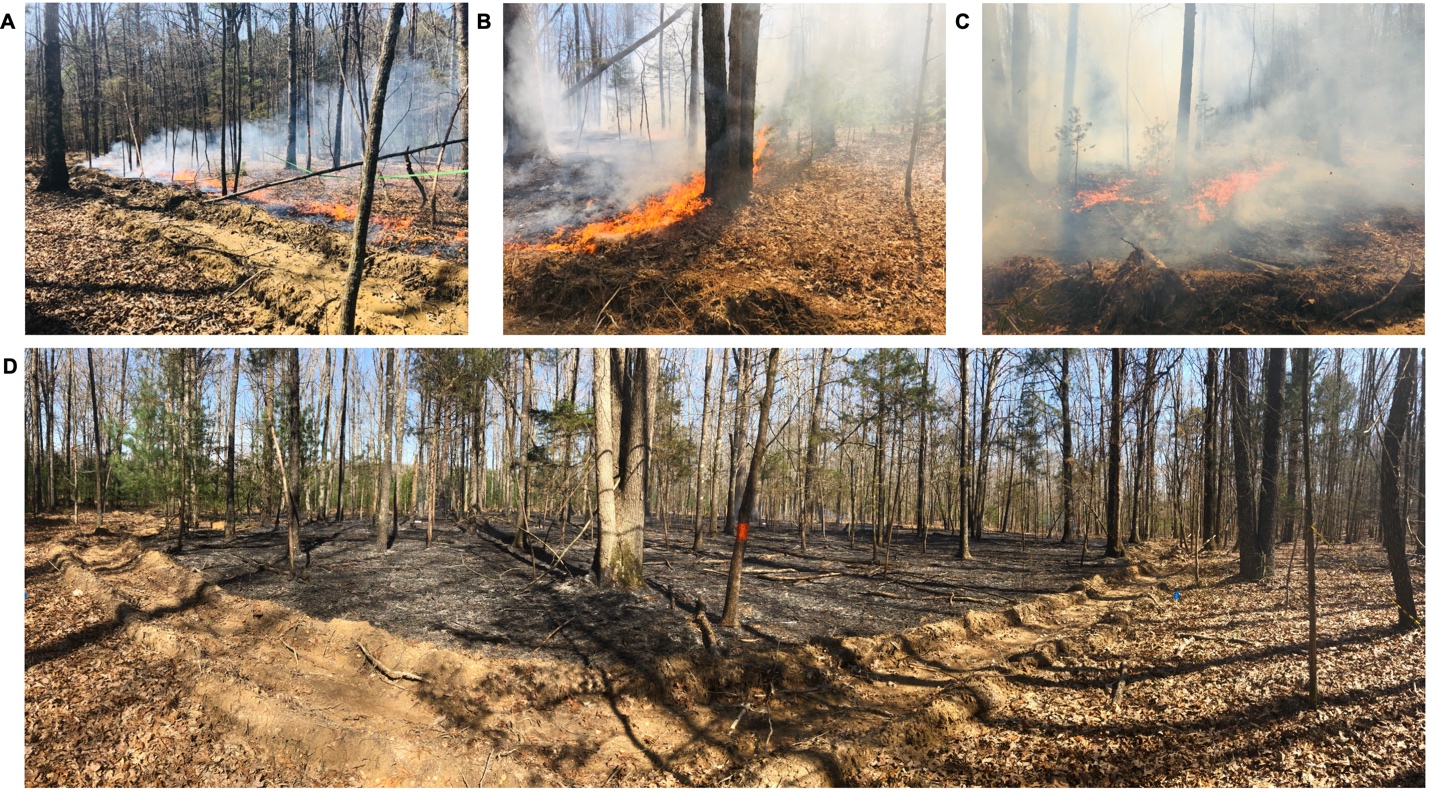
Figure S.1.** Prescribed burn pictures taken on 28 March 2019. (A) Litter ignited on the east side of the study site (B) Burn behaving as head fire (C) Burn behaving as back fire (D) Site after prescribed burn was completed.


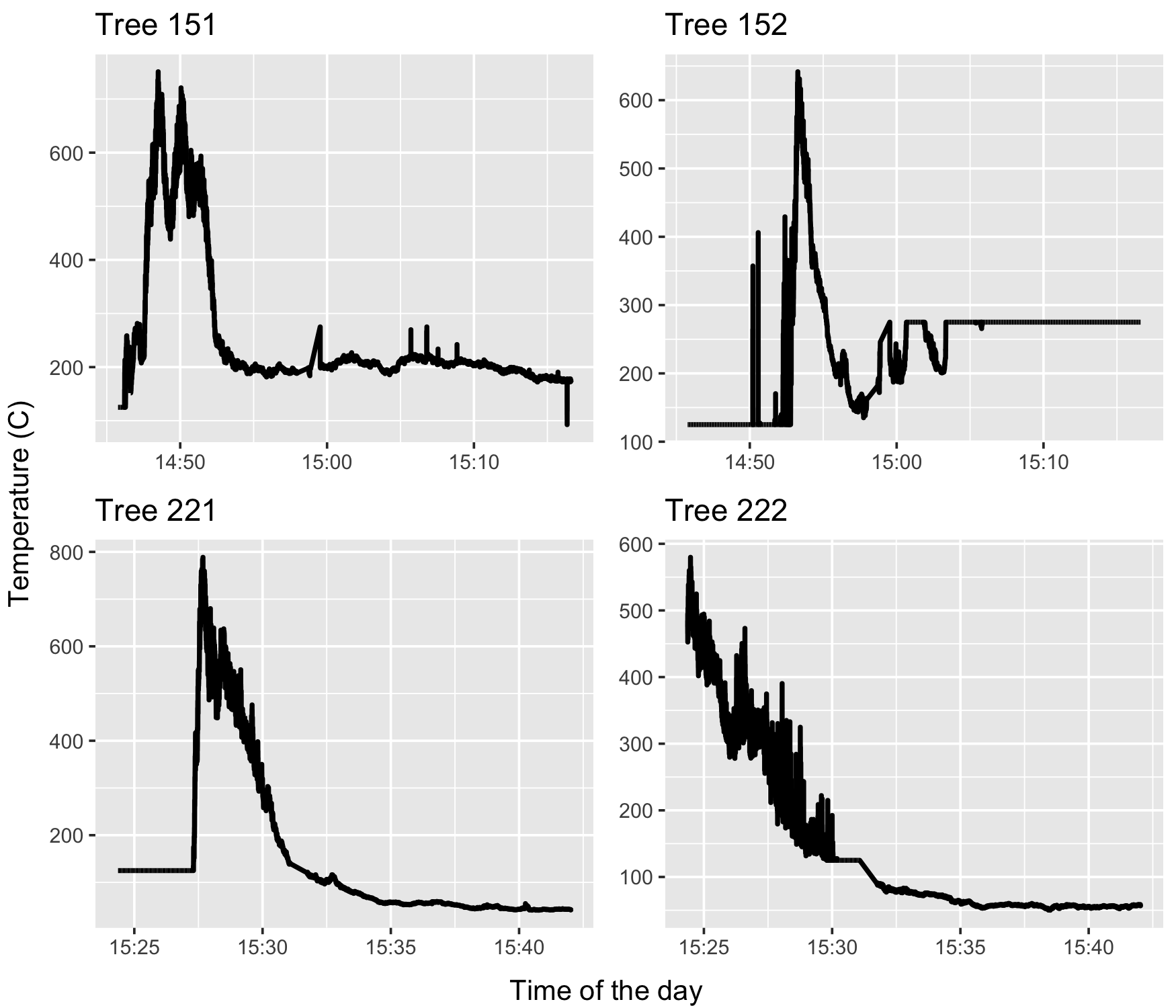


**Figure S.2.** Temperature of four of 10 flowering dogwood (*Cornus florida*) trees exposed to prescribed burn treatment. Measurements were taken using an infra-red imaging camera.


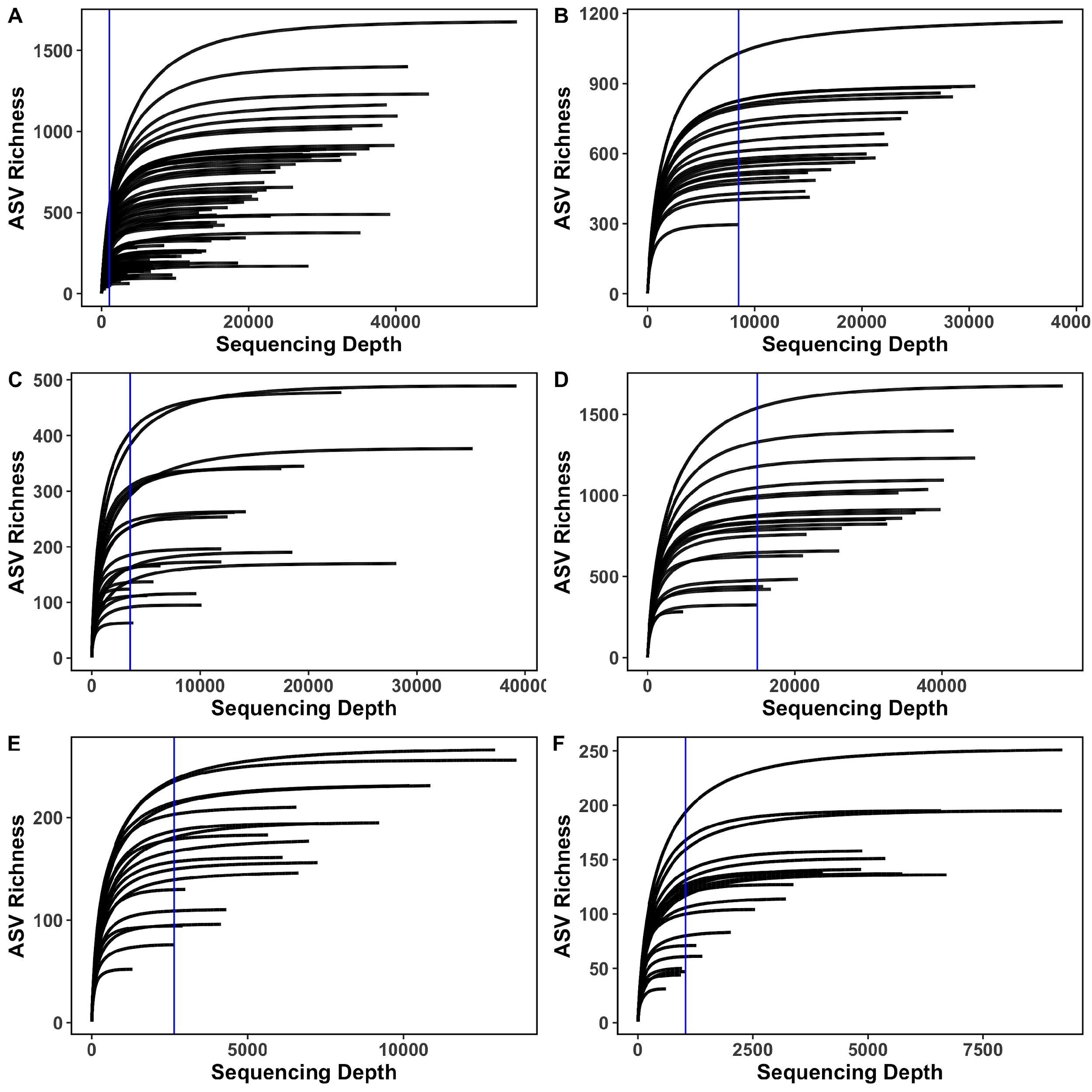


**Figure S.3.** Rarefaction curves for V3-V4 16S rRNA reads for (A) all niches, (B) bulk soils, (C) roots, (D) bark, (E) stem, and (F) leaves of flowering dogwood (*Cornus florida*) trees in the prescribed burn and unburned control treatments. Vertical blue lines represent the rarefaction cut-offs from table S.1.


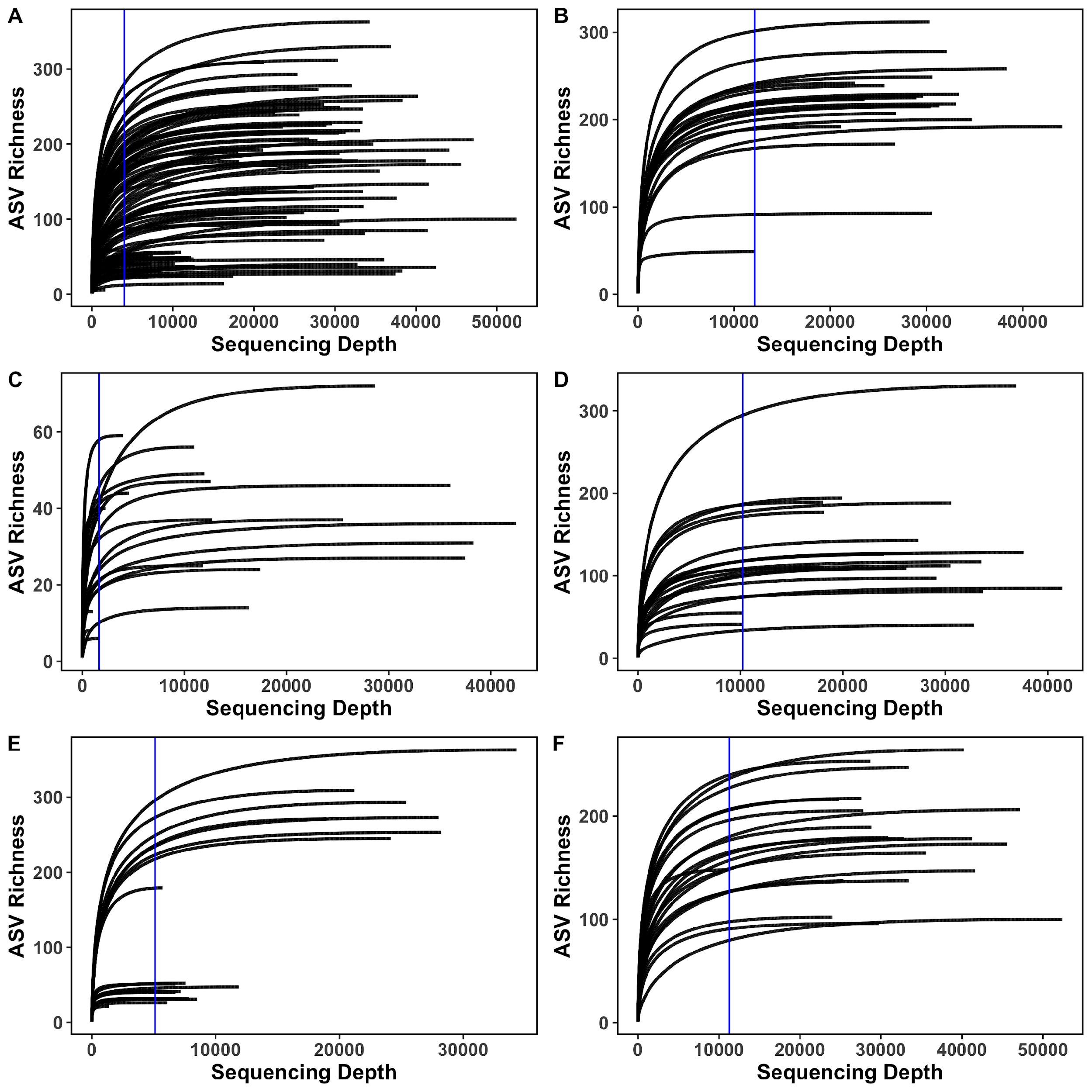


**Figure S.4.** Rarefaction curves for ITS2 sequences for (A) all niches, (B) bulk soils, (C) roots, (D) bark, (E) stem, and (F) leaves of flowering dogwood (*Cornus florida*) trees in the prescribed burn and unburned control treatments. Vertical blue lines represent the rarefaction cut-offs from table S.1.


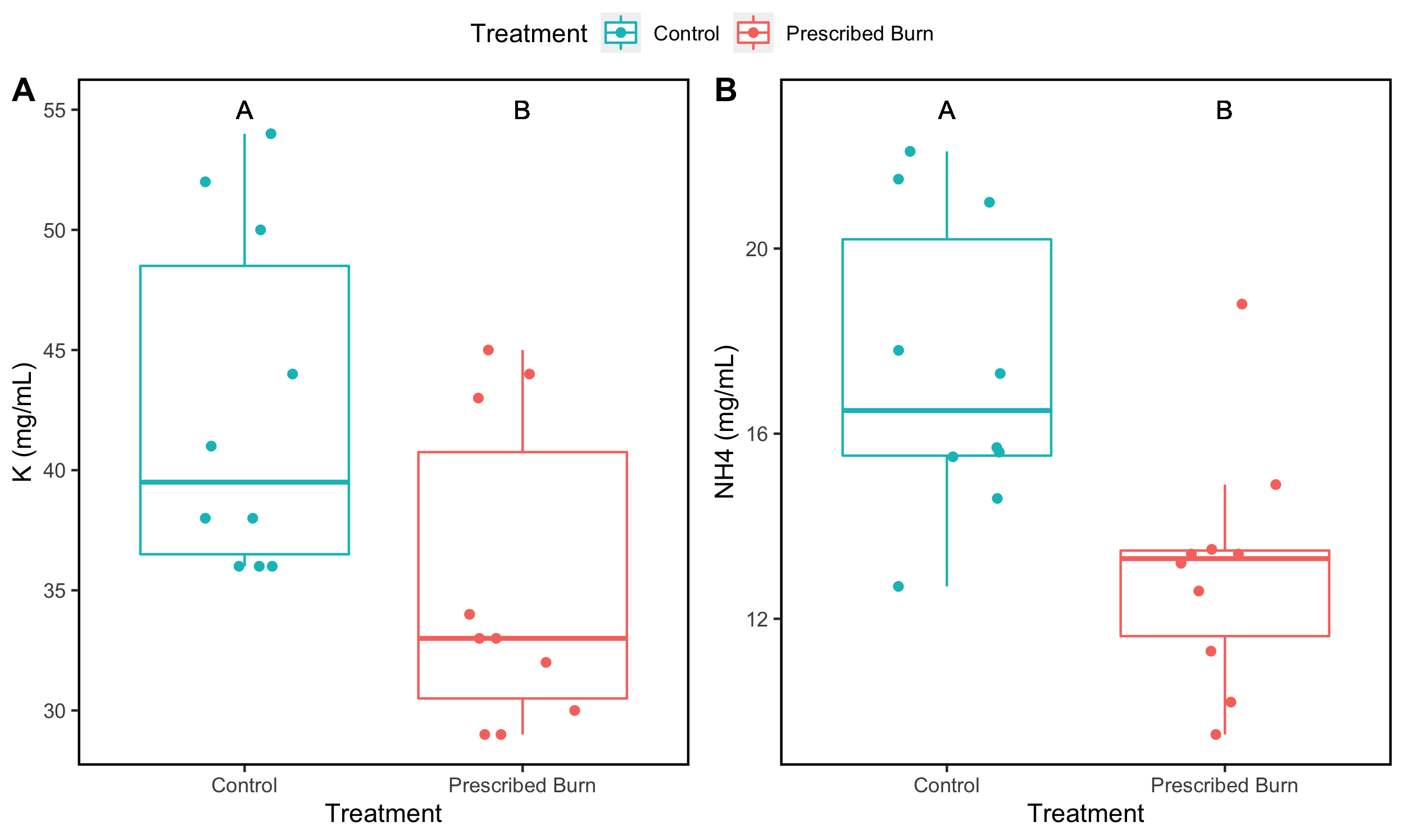


**Figure S.5.** Concentration of (A) potassium (K) and (B) ammonium (NH_4_) in soils collected from bases of flowering dogwood (*Cornus florida*) trees in prescribed burn and unburned control plots. Letters indicate significant mean differences determined using a two-sample *t*-test.


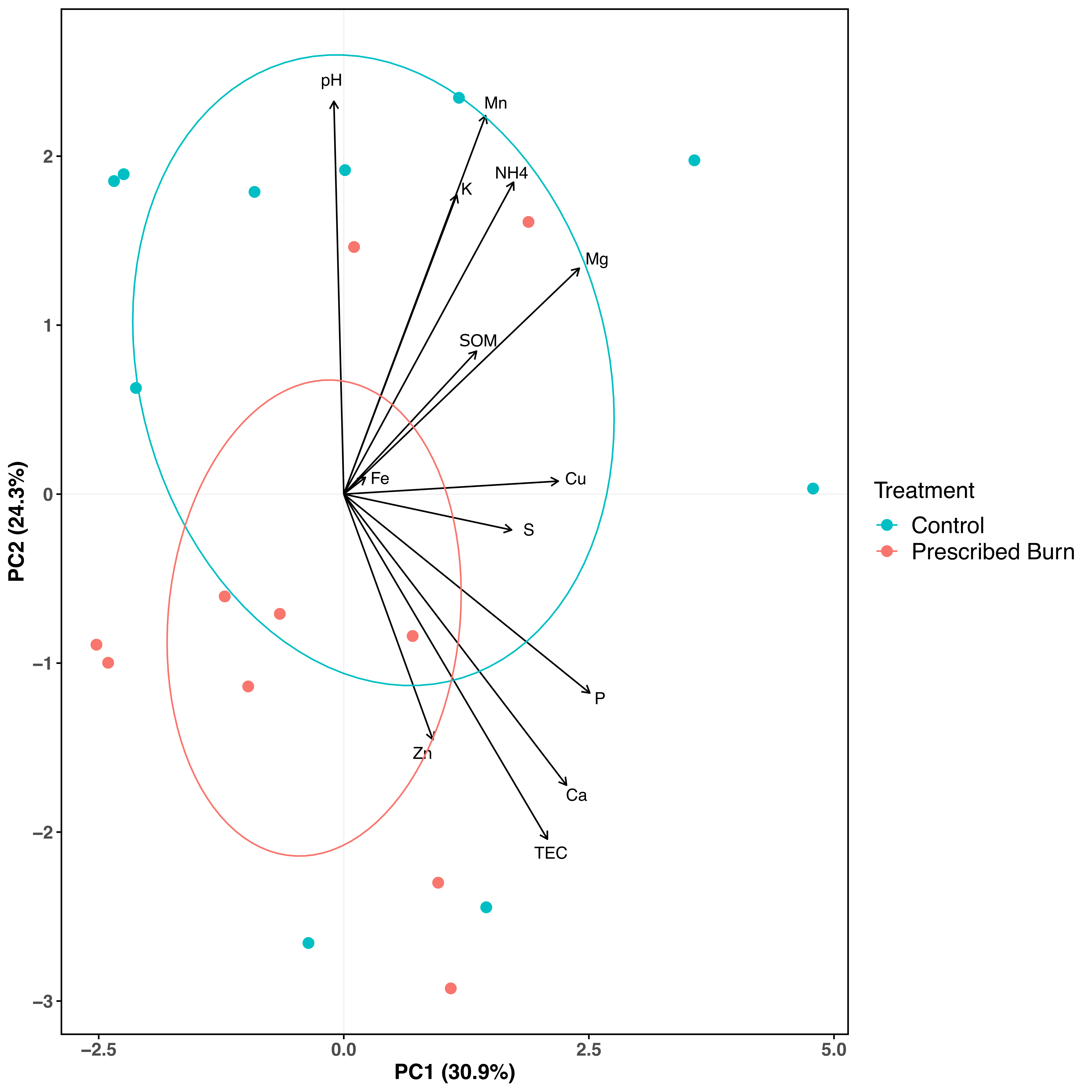


**Figure S.6. Principal component analysis (PCA) of soil physicochemical properties from unburned control and prescribed burn plots of flowering dogwood (*Cornus florida*) trees**. Color represents prescribed burn treatment. Ellipses represent standard deviation of axis scores from prescribed burn treatment centroids.
